# A Newly Secure Solution to MIMOME OFDM-Based SWIPT Frameworks: A Two-Stage Stackelberg Game for a Multi-User Strategy

**DOI:** 10.3390/e20010079

**Published:** 2018-01-22

**Authors:** Makan Zamanipour

**Affiliations:** Freelance Scholar, Tehran 1634776616, Iran; makan.zamanipour.2015@ieee.org

**Keywords:** MIMOME, OFDM, SWIPT

## Abstract

The paper technically proposes a newly secure scheme for simultaneous wireless power and information transfer (SWIPT) frameworks. We take into account an orthogonal frequency division multiplexing (OFDM)-based game which is in relation to a multi-input multi-output multi-antenna Eavesdropper (MIMOME) strategy. The transceiver is generally able to witness the case imperfect channel state information (ICSI) at the transmitter side. Transferring power and information are conducted via orthogonally provided sub-carriers. We propose a two-step Stackelberg game to optimise the Utility Functions of both power and information parts. The price for the first stage (in connection with information) is the total power of the other sub-carriers over which the energy is supported. In this stage, the sum secrecy rate should be essentially maximised. The second level of the proposed Stackelberg game is in association with the energy part. In this stage, the price essentially is the total power of the other sub-carriers over which the information is transferred. In this stage, additionally, the total power transferred is fundamentally maximised. Subsequently, the optimally and near-optimally mathematical solutions are derived, for some special cases such as ICSI one. Finally, the simulations validate our scheme as well, authenticating our contribution’s tightness and efficiency.

## 1. Introduction

Physical-layer security techniques basically play a vital role in Green communication these days. One of the important parts of these techniques are in relation to SWIPT frameworks [1,2,3]. These schemes as the emerging telecommunications technologies, are promising ones to highlight the term green cited above.

Two challenging issues as the open trends in connection with SWIPT systems are: (i) actualising a significantly appropriate tradeoff between the power and information parts; and (ii) guaranteeing the secrecy capacity.

In connection with the tradeoff expressed above, a coefficient (ratio) should be technically defined. Some recently conventional techniques to provide the mentioned tradeoff have been considered such as power splitting [4], time switching [5], antenna selection [3], full/half duplexing [4] etc. However, among them, one of the promising ones is sub-carriering [6,7,8,9,10,11,12].

The Stackelberg game has been widely taken into consideration in the literature aimed at principally guaranteeing physical layer security. In [13], a game was determined in which the transmitter was the Seller, whereas jammer was the Buyer. In [14,15,16], and even [17], the mentioned game-based method was considered as well in order to maintain the secrecy rate for cognitive radio frameworks. A dynamic hybrid-access control framework was defined in [18] exploiting the mentioned game. For energy harvesting schemes [19,20], some secure Stackelberg games have been provided to keep the quality of service.

### 1.1. Differences and Motivation (Regarding the Related Work)

Regarding the literature, optimally closed-form solutions in terms of jointly guaranteeing the secrecy capacity and power-information ratio are still required as favourable as possible. In other words, even if new solutions in the technologically advanced equipment SWIPT have been widely supported, however, they are also highly motivated.

More specifically, Ref. [21] sub-carriering in a MIMO OFDM-based SWIPT system in order to enhance the configuration flexibility. Indeed, in [21], the norm of each sub-channel was mathematically considered as 0.1× the number of the sub-carriers. In contrast, we use OFDM in order to pursue a goal. This paper technically discusses how to guarantee this goal.

Finally, it should be noted that classifying the resources to the two principles energy and information is often mapped to two totally different groups of users. Indeed, in the literature, there seems to be (i) some schemes [22,23] in which SWIPT is defined for every specific user; and (ii) some schemes [7,24,25,26,27,28] in which SWIPT is defined for two kinds of users, i.e., information decoders, and energy harvesters (which use their energy according to their disciplines). For example, Ref. [28] considered the energy/information for one class of users, whereas considering the artificial noise/energy for the others who were energy harvesters. Or even more specifically, Ref. [7] designated two separate classes of users, information receivers, and energy harvesters.

### 1.2. Our Contribution

More specifically, the contributions of our work are:

- Owing to the orthogonality between dually adjacent sub-carriers, the possibility of the energy receivers being passive Eavesdroppers (Eves) is zero. For example, in [12], an OFDM block was considered to jointly support the information and power. Inversely, in this paper, the coefficient to handle the power-information tradeoff is in relation to the number of sub-carriers. Instead, an active Eve is taken into account as well.
- We derive the mathematically closed-form solution as well.
- We extend and recast the solution into a sub-optimally closed-form solution using equal-power allocation.
- We extend the two-level Stackelberg to a stochastic one for the special case ICSI.

### 1.3. Notation & Organisation

Some of the symbols and mathematical notations used throughout the paper are shown in Table 1. Additionally, for more convenience, all vectors and matrices are respectively N×1 and N×N, without loss of optimality and generality.

The rest of the paper is organised as follows. Firstly, the main problem as well as the proposed solution are subsequently realised in Section 2. Additionally, the results are addressed in Section 3. Finally, conclusion and proofs are represented in Section 4 and Appendix A.

## 2. System Description and Problem Formulation

In this section, firstly the system and the main problem are described, subsequently, our proposed scheme is discussed.

### 2.1. System Description

**Figure 1:**
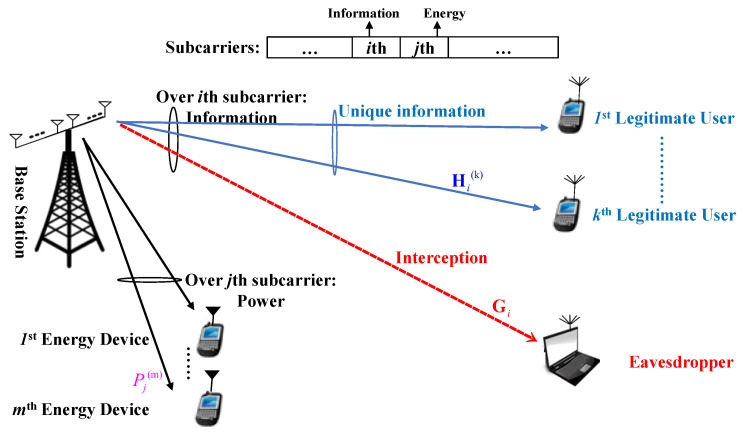
MIMOME OFDM-based Transceiver in downlink.

A MIMOME OFDM-based transceiver is given in Figure 1 supporting K legitimate users and M energy receivers. The MIMO channels between the legitimate transmitter and the *k*th legitimate user as well as the active Eve are formulated by $H^{\left(k\right)}$ and G (Our scheme unhesitatingly satisfies for H and G of sizes A×B,A≠B. However, we consider them square for more simplicity, without loss of generality and optimality.), in a downlink scenario. The superscripts *i* and *j* are relation to the sub-carriers over which the information and energy are respectively transferred. Moreover, due to the orthogonality between the pair of adjacent sub-carriers, the energy receivers cannot be passive Eves. Finally, the coefficient for handling the power-information tradeoff is in association with the number of sub-carriers.

We should strongly highlight here that our scheme does not experience any limitations. Though over both the *j*th sub-carrier, an energy harvester, and over the *i*th one, one information decoder is respectively allocated, however, the interception between these two adjacent sub-carriers is unified by zero. The interception should be fully considered since the energy harvester can potentially intercept the information decoder, due to the closer location to the access point. However, in order to guarantee a perfect scheme, an active Eavesdropper is also accordingly taken into account (We should add that classifying the resources to the two principles energy and information is often mapped to two totally different and separate sorts of users. Additionally, the energy harvested by the harvesters, may be used to decoding (such as in non-orthogonal multiple access scenarios in which we have two kinds of users, namely (i) strong users; and (ii) weak users); relaying (in cooperative schemes) etc.).

Assuming a fixed Bandwidth, Ergodic capacities $C_{r,i}^{\left(k\right)}$ and $C_{e,i}$ at the receiver of the *k*th legitimate user and Eve over the *i*th sub-carrier (*i*th sub-channel) are respectively considered as

(1) $$\begin{array}{c} \begin{array}{c} C_{r,i}^{\left(k\right)}=\log\left\{\det\left\{I+\frac{1}{\delta_{r,i}^{(k)2}}H_{i}^{\left(k\right)}Q_{i}H_{i}^{(k)H}\right\}\right\}, \end{array} \end{array}$$

and

(2) $$\begin{array}{c} \begin{array}{c} C_{e,i}=\log\left\{\det\left\{I+\frac{1}{\delta_{e,i}^{2}}G_{i}Q_{i}G_{i}^{H}\right\}\right\}, \end{array} \end{array}$$

with respect to (w.r.t.) the transmit covariance matrix $Q_{i}:=E\left\{x_{i}x_{i}^{H}\right\}$, and moreover the noise variance terms $\delta_{r,i}^{(k)2}$ and $\delta_{e,i}^{2}$ at the receiver sides (over the *i*th sub-channel). Thus, the secrecy capacity in relation to the *k*th user over the *i*th sub-carrier is defined as

(3) $$\begin{array}{c} \begin{array}{c} C_{S,i}^{\left(k\right)}:=max\left\{0,C_{r,i}^{\left(k\right)}-C_{e,i}\right\}. \end{array} \end{array}$$

**Fact 1:** Consider S, $A_{z}$ and $U_{z}$ respectively as a finite set of Players, a set of Actions of the *z*th Player, and the Utility Function for the *z*th Player. A Game $\left\{S,A_{z},U_{z}\right\}$ unhesitatingly has a Stackelberg Equilibrium, if

- $A_{z},\forall z\in Z$ is a non-empty compact convex subset over the Euclidian Space,
- and also $U_{z}$ is quasi-concave over $A_{z}$.

Please see [29] for the proof.

**Definition** **1** **(Stackelberg Equilibrium [30]).** *The vector of Action Space $A=\left({\tilde{a}}_{1},{\tilde{a}}_{2}\right)=\left({\tilde{a}}_{11},{\tilde{a}}_{12},{\tilde{a}}_{21},{\tilde{a}}_{22}\right)$ basically is a Stackelberg Equilibrium if and only if: ${\tilde{a}}_{1}=arg\;\underset{a_{1}}{max}U_{1}\left(a_{1},{\hat{a}}_{2}\left(a_{1}\right)\right)$ satisfies, where ${\hat{a}}_{2}\left(a_{1}\right)$$=arg\;\underset{a_{2}}{max}\left(a_{1},a_{2}\right),\forall a_{1}$, and also ${\tilde{a}}_{2}={\hat{a}}_{2}\left({\tilde{a}}_{1}\right).$*

**Definition** **2.** Consider a given Price set ${\left\{P_{n}^{*}\right\}}_{n=1}^{N},$n∈{i,j,i≠j,i=1,…,I,j=1,…,J},N∈{I,J,I≠J}. For example, $P_{i}^{*}$ is the power over the ith sub-carrier. As can be observed, the total number of sub-carriers is ρI+(1−ρ)J,∀ρ∈[0,1). ρ is a coefficient by which the option information/energy transferring is handled.

### 2.2. Our Two-Stage Stackelberg Game

Let us define the two-level Stackelberg Game G as

(4) $$\begin{array}{c} \begin{array}{c} G:\left\{\begin{array}{c} U_{info}:=\sum_{i=1}^{I}\sum_{k=1}^{K}C_{S,i}^{\left(k\right)}-\sum_{j=1,j\neq i}^{J}\sum_{m=1}^{M}P_{j}^{\left(m\right)},\\ U_{power}:=\sum_{j=1,j\neq i}^{J}\sum_{m=1}^{M}P_{j}^{\left(m\right)}-\sum_{i=1,i\neq j}^{I}P_{i}. \end{array}\right. \end{array} \end{array}$$

First step and the relative strategy: On this level, the Utility Function is $U_{info}$. The objective is the secrecy capacity for the *i*th sub-carrier for K legitimate users. The Price is the total transmit power of those sub-carriers over which power should be transferred to M energy receivers ($P_{j}^{\left(m\right)}$).

Second step and the relative strategy: On this level, the Utility Function is $U_{power}$. The objective is the total transmit power of those sub-carriers over which power should be transferred to M energy receivers. The Price is the total transmit power of those sub-carriers over which information should be transferred ($P_{i}$).

**Claim 1:** Our Stackelberg game is called two-stage since there appear to be two separately defined controllers, i.e., $P_{i}$ and $P_{j}^{\left(m\right)}$. These controllers are essentially assigned to respectively two separate group-Players legitimate users and energy receivers.

**Proposition** **1.** There undoubtedly exists a Stackelberg Equilibrium for the Game G.

**Proof.**  See Appendix A. ☐

**Theorem** **1.** *The Game G has an optimal solution as Problem $P_{1}$ as*

(5) $$\begin{array}{c} \begin{array}{c} \left(Q_{i}^{*},P_{i}^{*},P_{j}^{(m)*}\right)=arg\;\underset{Q_{i},P_{i},P_{j}^{\left(m\right)}}{max}\;\{\left\{\sum_{i=1}^{I}\sum_{k=1}^{K}C_{S,i}^{\left(k\right)}-\sum_{j=1,j\neq i}^{J}\sum_{m=1}^{M}P_{j}^{\left(m\right)}\right\},\\ \left\{\sum_{j=1,j\neq i}^{J}\sum_{m=1}^{M}P_{j}^{\left(m\right)}-\sum_{i=1,i\neq j}^{I}P_{i}\right\}\}\;\;, \end{array} \end{array}$$

*constraining*

(6) $$\begin{array}{c} \begin{array}{c} C_{1}:P_{i}:Tr\left\{Q_{i}Q_{i}^{H}\right\}\leq P_{th}^{\left(i\right)}, \end{array} \end{array}$$

*in which $P_{th}^{\left(i\right)}$ is a threshold defined for the transmit power over the ith sub-carrier, and finally*

(7) $$\begin{array}{c} \begin{array}{c} C_{2}:P_{j}^{\left(m\right)}\geq 0. \end{array} \end{array}$$

**Proof.**  See Appendix B. ☐

**Claim 2:** According to Claim 1, we should highlight that our Stackelberg game is called two-stage since, indeed, $Q_{i}^{*}\left(P_{i}^{*};P_{j}^{(m)*}\right)$ is a *2-D* controller w.r.t. both $P_{i}$ and $P_{j}^{\left(m\right)}$.

**Claim 3:** From (5), it can be conveniently witnessed that the security in our SWIPT scheme is efficiently guaranteed for the *k*th user, that is, $C_{S,i}^{\left(k\right)}$.

**Remark** **1.** *The Game G has a sub-optimal solution as Problem $P_{2}$ as*

(8) $$\begin{array}{c} \begin{array}{c} Q_{i}^{*}=arg\;\underset{Q_{i}}{max}\left\{\left\{\sum_{i=1}^{I}\sum_{k=1}^{K}C_{S,i}^{\left(k\right)}-\sum_{j=1,j\neq i}^{J}\sum_{m=1}^{M}P_{j}^{\left(m\right)}\right\},\left\{\sum_{j=1,j\neq i}^{J}\sum_{m=1}^{M}P_{j}^{\left(m\right)}-\sum_{i=1,i\neq j}^{I}P_{i}\right\}\right\}, \end{array} \end{array}$$

*while constraining*

(9) $$\begin{array}{c} \begin{array}{c} C_{1}:P_{i}:Tr\left\{Q_{i}Q_{i}^{H}\right\}\leq \frac{1}{\rho I}, \end{array} \end{array}$$

*and finally*

(10) $$\begin{array}{c} \begin{array}{c} C_{2}:P_{j}^{\left(m\right)}\leq \frac{1}{(1-\rho )\mathrm{JM}}. \end{array} \end{array}$$

**Proposition** **2.** *Equivalently, Problem $P_{2}$ can also be consequently re-stated as Problem $P_{3}$ as*

(11) $$\begin{array}{c} \begin{array}{c} Q_{i}^{*}=arg\;\underset{Q_{i}}{max}\left\{\left\{\sum_{i=1}^{I}\sum_{k=1}^{K}C_{S,i}^{\left(k\right)}-\frac{1}{(1-\rho )\mathrm{JM}}\right\},\left\{\frac{1}{(1-\rho )\mathrm{JM}}-\frac{1}{\rho I}\right\}\right\}. \end{array} \end{array}$$

**Proof.**  Regarding the principle Equal-power allocation, Problem $P_{1}$ can be conveniently re-casted into Problem $P_{3}$, which near-optimally satisfies. ☐

**Remark** **2.** Generally, assuming equal-power allocation among the sub-carriers consequently reduces the energy consumption at the transmitter and receiver, as completely discussed in [12].

**Remark** **3.** The rates obtained through two totally different approaches Water-filling and equal-power allocation for the sub-carriers are nearly equal [12,31].

**Proposition** **3** **(ICSI case).** *Under the assumption of experiencing the case ICSI, the Game G has another sub-optimal solution as Problem $P_{4}$ as*

(12) $$\begin{array}{c} \begin{array}{c} \;\underset{Q_{i}}{max}\;R_{i}^{\left(k\right)}, \end{array} \end{array}$$

*while consequently defining the following probabilistic constraint w.r.t. $C_{S,i}^{\left(k\right)}\in \left[0,R_{i}^{\left(k\right)}\right)$*

(13) $$\begin{array}{c} \begin{array}{c} C_{1}:P\left\{C_{S,i}^{\left(k\right)}\geq R_{i}^{\left(k\right)}\right\}\geq 1-\epsilon , \end{array} \end{array}$$

*and*

(14) $$\begin{array}{c} \begin{array}{c} C_{2}:P_{i}:Tr\left\{Q_{i}Q_{i}^{H}\right\}\leq \frac{1}{\rho I}, \end{array} \end{array}$$

*and finally*

(15) $$\begin{array}{c} \begin{array}{c} C_{3}:P_{j}^{\left(m\right)}\leq \frac{1}{(1-\rho )\mathrm{JM}}, \end{array} \end{array}$$

*in which (13), according to Bernstein inequality as a probabilistic method to deal with the controversial case ICSI (see for example [32,33]), can be re-casted as*

(16) $$\begin{array}{c} \begin{array}{c} \left\{\begin{array}{c} \;Tr\left\{G_{i}^{\frac{1}{2}}F_{i}^{\left(k\right)}G_{i}^{\frac{1}{2}}\right\}-\sqrt{-2\ln(\epsilon )}\alpha_{i}^{\left(k\right)}+\ln\left(\epsilon \right)\beta_{i}^{\left(k\right)}+d_{1,i}^{\left(k\right)}\geq 0,\\ \|\left[\begin{array}{c} vec\left(G_{i}^{\frac{1}{2}}F_{i}^{\left(k\right)}G_{i}^{\frac{1}{2}}\right)\\ {\sqrt{2}G_{i}^{\frac{1}{2}}F_{i}^{\left(k\right)}g}_{i} \end{array}\right]\|\leq \alpha_{i}^{\left(k\right)},\;\;\;\;\;\;\;\;\;\;\\ \;\beta_{i}^{\left(k\right)}I_{N}+G_{i}^{\frac{1}{2}}F_{i}^{\left(k\right)}G_{i}^{\frac{1}{2}}\geq 0, \end{array}\right. \end{array} \end{array}$$

*where $\alpha_{i}^{\left(k\right)}$ and $\beta_{i}^{\left(k\right)}$ are Slack variables which are derived by the algorithm given in Algorithm 1 w.r.t. DogLeg method [34,35], and*

(17) $$\begin{array}{c} \begin{array}{c} d_{1,i}^{\left(k\right)}:=g_{i}^{H}F_{i}^{\left(k\right)}g_{i}-\left(\left(2^{\left(R_{i}^{\left(k\right)}\right)}\right)d_{2,i}^{\left(k\right)}-1\right)\delta_{e,i}^{2}, \end{array} \end{array}$$

(18) $$\begin{array}{c} \begin{array}{c} F_{i}^{\left(k\right)}:=-2^{\left(R_{i}^{\left(k\right)}\right)}d_{2,i}^{\left(k\right)}Q_{i}, \end{array} \end{array}$$

*and*

(19) $$\begin{array}{c} \begin{array}{c} d_{2,i}^{\left(k\right)}:=\frac{\delta_{r,i}^{(k)2}}{h_{i}^{(k)H}Q_{i}h_{i}^{\left(k\right)}}, \end{array} \end{array}$$

*w.r.t.*

(20) $$\begin{array}{c} \begin{array}{c} {\hat{g}}_{i}:=g_{i}+\underset{\Delta g_{i}}{\underset{︸}{G_{i}^{\frac{1}{2}}g_{i}}}, \end{array} \end{array}$$

*and*

(21) $$\begin{array}{c} \begin{array}{c} \left\{\begin{array}{c} g_{i}:=vec\left\{G_{i}\right\},\\ h_{i}^{\left(k\right)}:=vec\left\{H_{i}^{\left(k\right)}\right\}. \end{array}\right. \end{array} \end{array}$$

**Algorithm 1.** Dogleg method [34,35]. Proposed algorithm for deriving $\alpha_{i}^{\left(k\right)}$ and $\beta_{i}^{\left(k\right)}$.Output: $\alpha_{i}^{(k)*}$, $\beta_{i}^{(k)*}$**1. Initialisation:** Set the Knees $\varphi_{i}^{\left(k\right)}\left(\alpha ;t=0\right),\varphi_{i}^{\left(k\right)}\left(\beta ;t=0\right),$ and the Legs $\gamma_{i}^{\left(k\right)}\left(\varphi ,\alpha ;t=0\right),$
$\gamma_{i}^{\left(k\right)}\left(\varphi ,\beta ;t=0\right)$.**2. Computation:** Until convergence**3. Repeat:** Update the Knees and Legs; go to Step 2; t=t+1.

## 3. Results and Evaluation

In this section, we provide a theoretical and simulation-based analysis over our newly proposed framework.

Some parameters taken into account in the simulations are given in Table 2, all of which are fixed, if not mentioned otherwise.

**Figure 2:**
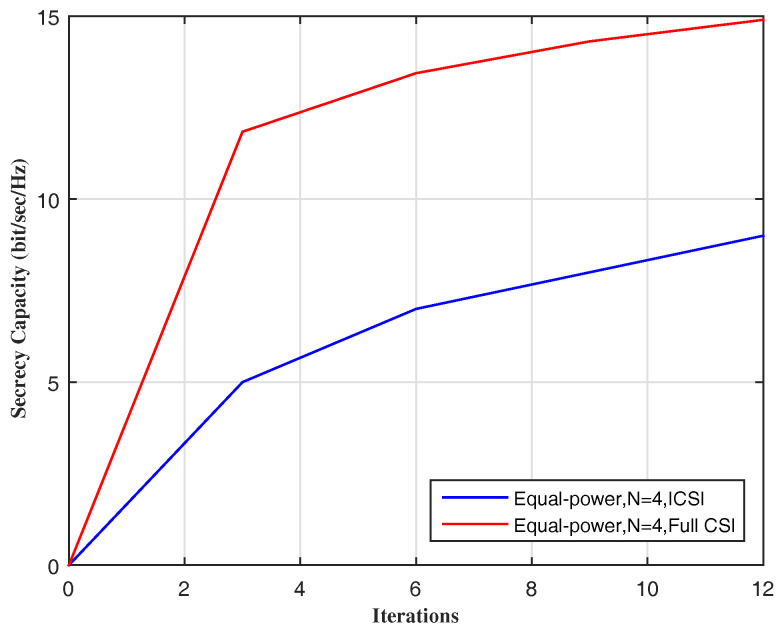
Secrecy capacity vs. iterations’ number.

The secrecy capacity is depicted in Figure 2 against the number of iterations, for the two cases ICSI and full CSI, where the size of MIMO, i.e., *N* is 4, using the equal-power technique.

**Figure 3:**
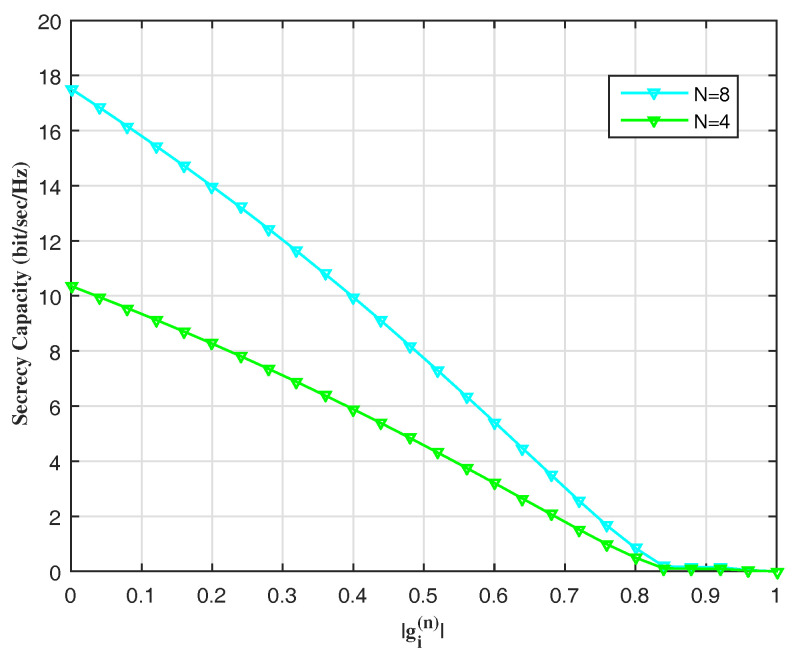
Secrecy capacity vs. the interception channel’s norm.

The secrecy capacity is shown in Figure 3 against the norm of the interception channel, while changing the size of MIMO, i.e., *N*. In this figure, an average is thoroughly applied on *n* entries of the matrices.

**Remark** **4.** It should be noted that Figure 3 succinctly indicates the Eve’s performance.

**Figure 4:**
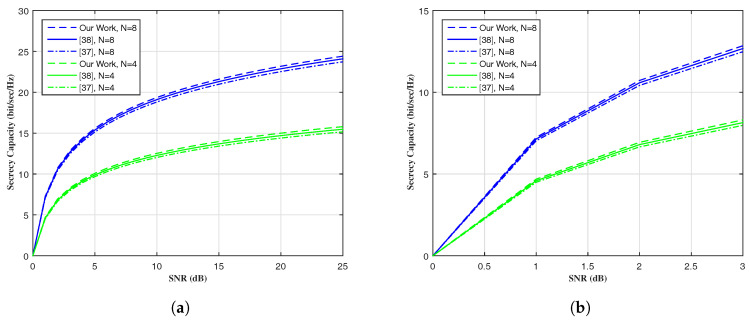
Secrecy capacity vs. SNR(dB). (**a**) Complete SNR(dB) regime; (**b**) Low SNR(dB) regime.

Figure 4 shows the secrecy capacity versus SNR(dB) while changing the size of MIMO, i.e., *N*, comparing our work with [37,38]. Our proposed algorithm performs better than [37,38].

**Figure 5:**
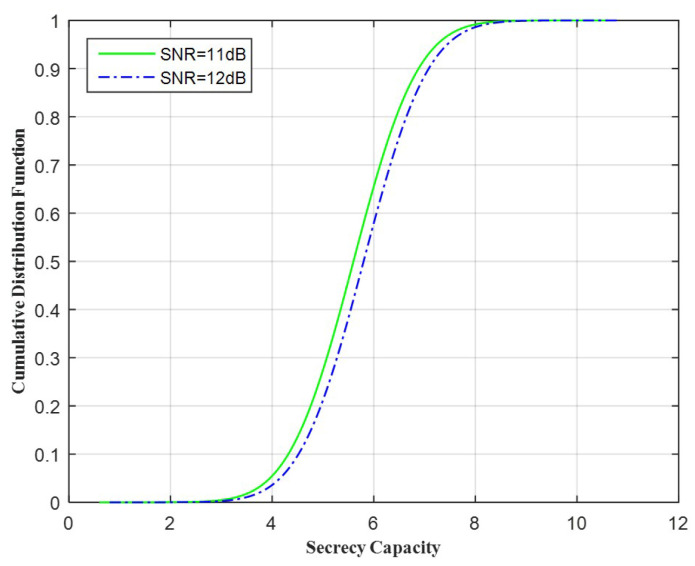
Cumulative distribution function vs. the secrecy capacity.

Figure 5 shows the Cumulative distribution function of the secrecy capacity while changing SNR, for the case ICSI.

**Figure 6:**
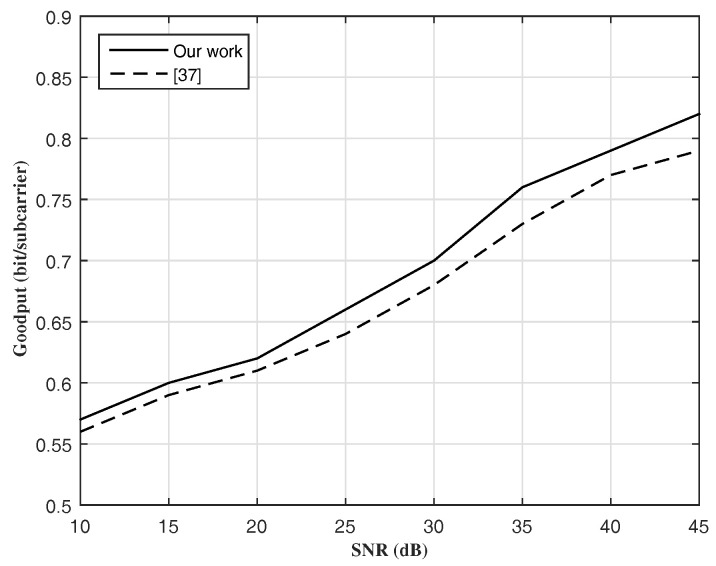
Goodput vs. SNR(dB).

Figure 6 illustrates the principle Goodput as the spent bits per sub-carrier (as the useful data: the size of a transmitted packet devided to the transfer time), while comparing our work and [38]. Our proposed algorithm performs better than [38].

The proposed algorithm to derive the best solution to our proposed scheme is given in Algorithm 2.

**Lemma** **1.** The algorithm stated above converges.

**Proof.**  The algorithm is descending due to the decreasing behaviour of the inputs in the iteration step, when $Q_{i}\left(P_{i};P_{j}^{\left(m\right)}\right)\to Q_{i}^{*}\left(P_{i}^{*};P_{j}^{(m)*}\right)$, hence, this converges. The inputs are assigned from the relative equations, as shown in the algorithm, i.e., $P_{th}^{\left(i\right)},\rho ,I,J,M,\epsilon ,\alpha_{i}^{\left(k\right)},\beta_{i}^{\left(k\right)}$. ☐

**Algorithm 2.** Proposed Resource Scheduling Algorithm.**Intput:**
$P_{th}^{\left(i\right)},\rho ,I,J,M,\epsilon ,\alpha_{i}^{\left(k\right)},\beta_{i}^{\left(k\right)}$**Output:**
$Q_{i}^{*}\left(P_{i}^{*};P_{j}^{(m)*}\right)$**1. Initialisation:** Set the given thresholds, counter=0.**2. Computation:** Until convergence **Switch**   **Case 1**
*Perfect CSI*       Solve Problem $P_{1}$ (or $P_{3}$).   **Case 2**
*Imperfect CSI*       Solve Problem $P_{4}$. **end****If**
Q* is feasible for Problem, stop; store the resultant matrix.**3. Iteration:** Repeat**Else if** Otherwise, empty Q*; re-assign the possible candidates; update the subsets; go to Step 2; counter=counter+1.**End**

As completely discussed in [37], since the utility functions are tightly concave over the feasible solution sets, irrespective of some rare cases (for example spatially correlated MIMOs for which other interface algorithms has been proposed such as proximal-point-based regularization approach [37]), the algorithm converges. Of course, the aforementioned concavity is proven in the next parts, as well.

**Lemma** **2.** **(Complexity):** Our scheme’s complexity in terms of Big-O function is ≈$O(N^{3})$, compared to ≈$O(N^{5})$ obtained for [38].

The figures and discussions mentioned above straightforwardly highlight our scheme’s efficiency and correctness.

## 4. Conclusions

A secure scheme for SWIPT systems was proposed in a MIMOME OFDM-multi-user-based strategy. OFDM was used to transfer the power and information. A two-level Stackelberg game was also proposed to optimise the Utility Functions of both power and information sides. The novelty stated above was conducted as well, supporting some theorems and propositions.

## Figures and Tables

**Table 1:** Notations.

Notation	Definition	Notation	Definition
A	Matrix	log{·}	Logarithm
a	Vector	Tr{·}	Trace of Matrix
*a*	Scalar	max{·}	Maximum Value
I	Identity Matrix	Sup	Supremum
**0**	All-zero Matrix	vec{·}	Vectorisation
$\hat{\left(\cdot \right)}$	Estimation operation	‖·‖	Euclidean Norm
${\left\{\cdot \right\}}^{H}$	Hermitian	:=	Equal by Definition
det{·}	Determinant	${\left(\cdot \right)}^{*}$	Optimum Value

**Table 2:** Simulation parameters.

Parameter	Value
The size of MIMOs (N)	4 (i.e., 4 × 4)
Transmit power threshold $P_{th}^{\left(i\right)}$	SNR regime
Convex interface package	CVX [36]
Number of sub-carriers	1024
Number of legitimate users (*k*)	25
Number of Randomly generated channel realisations	300

## Appendix A

### Proof of Proposition 1

The existence of any Stackelberg Equilibrium for the Game G is proven according to the uniqueness of the Game G. Prior of this, we should prove the mentioned uniqueness regarding the concavity of the two-step Game G. The concavity expressed above is proven regarding the fact that both steps of the Game G are concave. Please see the following.

It is sufficient to prove the concavity (see for example [39]) of only the first level. $h(bx_{1}+\left(1-b\right)x_{2})$, 0≤b≤1 is concave if and only if $\frac{\partial^{2}h\left(b\right)}{\partial b^{2}}\leq 0$ (see for example [40]). Now, let us define the function (For more convenience, in order to simplify the following equations (only in Appendix A), please consider a single-user case. This assumption results in removing the superscript *k* in the following).

(A1) $$\begin{array}{c} \begin{array}{c} h\left(Q_{i}\right):=\log\left\{\det\left\{I+\frac{1}{\delta_{r,i}^{2}}H_{i}Q_{i}H_{i}^{H}\right\}\right\}-\log\left\{\det\left\{I+\frac{1}{\delta_{e,i}^{2}}G_{i}Q_{i}G_{i}^{H}\right\}\right\},\;\;\; \end{array} \end{array}$$

and

(A2) $$\begin{array}{c} \begin{array}{c} Q_{i}^{\left(2\right)}+b\left(Q_{i}^{\left(2\right)}-Q_{i}^{\left(1\right)}\right)=U_{i}+bV_{i}, \end{array} \end{array}$$

therefore,

(A3) $$\begin{array}{c} \begin{array}{c} h_{1}\left(b\right)=\log\left\{\det\left\{I+\left(U_{i}^{\left(1\right)}+bV_{i}^{\left(1\right)}\right)W_{i}^{\left(1\right)}\right\}\right\}, \end{array} \end{array}$$

and

(A4) $$\begin{array}{c} \begin{array}{c} h_{2}\left(b\right)=\log\left\{\det\left\{I+\left(U_{i}^{\left(2\right)}+bV_{i}^{\left(2\right)}\right)W_{i}^{\left(2\right)}\right\}\right\}, \end{array} \end{array}$$

where

(A5) $$\begin{array}{c} \begin{array}{c} W_{i}^{\left(1\right)}:=H_{i}^{H}H_{i}, \end{array} \end{array}$$

(A6) $$\begin{array}{c} \begin{array}{c} W_{i}^{\left(2\right)}:=G_{i}^{H}G_{i}. \end{array} \end{array}$$

Let us define new variables

(A7) $$\begin{array}{c} \begin{array}{c} X_{i}^{\left(1\right)}:=I+U_{i}^{\left(1\right)}W_{i}^{\left(1\right)}, \end{array} \end{array}$$

(A8) $$\begin{array}{c} \begin{array}{c} X_{i}^{\left(2\right)}:=I+U_{i}^{\left(2\right)}W_{i}^{\left(2\right)}, \end{array} \end{array}$$

(A9) $$\begin{array}{c} \begin{array}{c} Y_{i}^{\left(1\right)}:=V_{i}^{\left(1\right)}W_{i}^{\left(1\right)}, \end{array} \end{array}$$

(A10) $$\begin{array}{c} \begin{array}{c} Y_{i}^{\left(2\right)}:=V_{i}^{\left(2\right)}W_{i}^{\left(2\right)}. \end{array} \end{array}$$

Hence,

(A11) $$\begin{array}{c} \begin{array}{c} h_{f}\left(b\right):=\log\left\{\det\left\{X_{i}^{\left(f\right)}+bY_{i}^{\left(f\right)}\right\}\right\},f\in \left\{1,2\right\}, \end{array} \end{array}$$

which is equal to

(A12) $$\begin{array}{c} \begin{array}{c} h_{f}\left(b\right)=\log\left\{\det\left\{X_{i}^{\left(f\right)\frac{1}{2}}\left(I+b\underset{D_{i}^{\left(f\right)}}{\underset{︸}{X_{i}^{\left(f\right)\frac{-1}{2}}Y_{i}^{\left(f\right)}X_{i}^{\left(f\right)\frac{-1}{2}}}}\right)X_{i}^{\left(f\right)\frac{1}{2}}\right\}\right\}=\\ \sum_{d=1}^{D}\log\left(1+b\lambda_{d}^{\left(f\right)}\right)+\log\left\{\det\left\{X_{i}^{\left(f\right)}\right\}\right\},f\in \left\{1,2\right\},\;\; \end{array} \end{array}$$

for which $\lambda_{d}^{\left(f\right)}$ are the eigen-values of $D_{i}^{\left(f\right)},\;\forall f\in \left\{1,2\right\}$.

Now, we have

(A13) $$\begin{array}{c} \begin{array}{c} \frac{\partial^{2}h_{f}\left(b\right)}{\partial b^{2}}=-\sum_{d=1}^{D}\frac{\lambda_{d}^{2(f)}}{{\left(1+b\lambda_{d}^{\left(f\right)}\right)}^{2}}\leq 0,f\in \left\{1,2\right\}. \end{array} \end{array}$$

Thus, the proof is completed regarding the concavity derived above.

## Appendix B

### Proof of Theorem 1

First of all, let us provide the following definition [41].

**Definition** **A1.** *For the Stackelberg Equilibrium $\left(a_{1},a_{2}\right)$ the following should hold*

(A14) $$\begin{array}{c} \begin{array}{c} U_{z}\left(a_{1}\right)=\underset{\underset{a_{z}}{Sup}}{Sup}U_{z}\left(a_{z}^{\left(1\right)}\right), \end{array} \end{array}$$

*and*

(A15) $$\begin{array}{c} \begin{array}{c} U_{z}\left(a_{2}\right)=\underset{\underset{a_{z}}{Sup}}{Sup}U_{z}\left(a_{z}^{\left(2\right)}\right). \end{array} \end{array}$$

Regarding Definition A1 given above, now, the following equations hold

(A16) $$\begin{array}{c} \begin{array}{c} \sum_{i=1}^{I}\sum_{k=1}^{K}C_{S,i}^{\left(k\right)}\left(Q_{i}^{*};P_{i}^{*}\right)-\sum_{j=1,j\neq i}^{J}\sum_{m=1}^{M}P_{j}^{\left(m\right)}\geq \sum_{i=1}^{I}\sum_{k=1}^{K}C_{S,i}^{\left(k\right)}\left(Q_{i};P_{i}^{*}\right)-\sum_{j=1,j\neq i}^{J}\sum_{m=1}^{M}P_{j}^{\left(m\right)}, \end{array} \end{array}$$

(A17) $$\begin{array}{c} \begin{array}{c} \sum_{i=1}^{I}\sum_{k=1}^{K}C_{S,i}^{\left(k\right)}\left(Q_{i}^{*};P_{i}^{*}\right)-\sum_{j=1,j\neq i}^{J}\sum_{m=1}^{M}P_{j}^{\left(m\right)}\geq \sum_{i=1}^{I}\sum_{k=1}^{K}C_{S,i}^{\left(k\right)}\left(Q_{i}^{*};P_{i}\right)-\sum_{j=1,j\neq i}^{J}\sum_{m=1}^{M}P_{j}^{\left(m\right)}, \end{array} \end{array}$$

(A18) $$\begin{array}{c} \begin{array}{c} \sum_{i=1}^{I}\sum_{k=1}^{K}C_{S,i}^{\left(k\right)}\left(Q_{i}^{*};P_{i}\right)-\sum_{j=1,j\neq i}^{J}\sum_{m=1}^{M}P_{j}^{\left(m\right)}\geq \sum_{i=1}^{I}\sum_{k=1}^{K}C_{S,i}^{\left(k\right)}\left(Q_{i};P_{i}\right)-\sum_{j=1,j\neq i}^{J}\sum_{m=1}^{M}P_{j}^{\left(m\right)}, \end{array} \end{array}$$

(A19) $$\begin{array}{c} \begin{array}{c} \sum_{i=1}^{I}\sum_{k=1}^{K}C_{S,i}^{\left(k\right)}\left(Q_{i};P_{i}^{*}\right)-\sum_{j=1,j\neq i}^{J}\sum_{m=1}^{M}P_{j}^{\left(m\right)}\geq \sum_{i=1}^{I}\sum_{k=1}^{K}C_{S,i}^{\left(k\right)}\left(Q_{i};P_{i}\right)-\sum_{j=1,j\neq i}^{J}\sum_{m=1}^{M}P_{j}^{\left(m\right)}, \end{array} \end{array}$$

and

(A20) $$\begin{array}{c} \begin{array}{c} \sum_{j=1,j\neq i}^{J}\sum_{m=1}^{M}P_{j}^{(m)*}-\sum_{i=1,i\neq j}^{I}P_{i}\geq \sum_{j=1,j\neq i}^{J}\sum_{m=1}^{M}P_{j}^{\left(m\right)}-\sum_{i=1,i\neq j}^{I}P_{i}. \end{array} \end{array}$$

Hence, we are able to proceed with

(A21) $$\begin{array}{c} \begin{array}{c} \frac{\partial \left\{C_{S,i}^{\left(k\right)}\left(Q_{i}^{*};P_{i}\right)\right\}}{\partial Q_{i}^{*}}=0, \end{array} \end{array}$$

As completely discussed for example in [33] (Equation (43)) can be conveniently derived exploiting the facts $\partial \left\{\log\left\{det\left\{U\right\}\right\}\right\}=Tr\left\{U^{-1}\partial U\right\}$ [42], chain rule as $\frac{\partial g(U=f(U))}{\partial U}$ [43] (Equation (135)), and $\frac{\partial (W(U)V)}{\partial U}=\left(\partial \left(W\left(U\right)\right)\right)V+0$ [43] (Equation (37)).

Solving (42) completes the proof.

## References

[1] Wang X., Liu J., Zhai C., Ma S., Wang Q. (2016). Energy efficient relay networks with wireless power transfer from a multi-antenna base station. Trans. Emerg. Telecommun. Technol..

[2] Liu C., Natarajan B. (2017). Modeling and Analysis of Simultaneous Information and Energy Transfer in Internet of Things. Trans. Emerg. Telecommun. Technol..

[3] Zahedi A., Lari M., Albaaj A., Alabkhat Q. (2017). Simultaneous energy harvesting and information processing considering multi-relay multi-antenna using maximum ratio transmission and antenna selection strategies. Trans. Emerg. Telecommun. Technol..

[4] Dong A., Zhang H., Shu M., Yuan D. (2017). Simultaneous Wireless Information and Power Transfer for MIMO Interference Channel Networks Based on Interference Alignment. Entropy.

[5] Pan G., Tang C. (2017). Outage Performance on Threshold AF and DF Relaying Schemes in Simultaneous Wireless Information and Power Transfer Systems. Int. J. Electron. Commun..

[6] Huang X., Li Q., Zhang Q., Qin J. (2014). Power allocation for secure OFDMA systems with wireless information and power transfer. Electron. Lett..

[7] Zhang M., Liu Y., Zhang R. (2016). Artificial Noise Aided Secrecy Information and Power Transfer in OFDMA Systems. IEEE Trans. Wirel. Commun..

[8] Zhang M., Liu Y. (2016). Energy Harvesting for Physical-Layer Security in OFDMA Networks. IEEE Trans. Inf. Forensics Secur..

[9] Li M., Liu Y. (2017). Power Allocation for Secure SWIPT Systems with Wireless-Powered Cooperative Jamming. IEEE Commun. Lett..

[10] Zhang M., Liu Y., Zhang R. Secrecy Wireless Information and Power Transfer in OFDMA Systems. Proceedings of the 2015 IEEE Global Communications Conference (GLOBECOM).

[11] Zhang M., Liu Y., Feng S. Energy Harvesting for Secure OFDMA Systems. Proceedings of the 2014 Sixth International Conference on Wireless Communications and Signal Processing (WCSP).

[12] Shafie A.E., Tourki K., Al-Dhahir N.L. (2017). An Artificial-Noise-Aided Hybrid TS/PS Scheme for OFDM-Based SWIPT Systems. IEEE Commun. Lett..

[13] Tang X., Ren P., Wang Y., Du Q., Sun L. Securing Wireless Transmission against Reactive Jamming: A Stackelberg Game Framework. Proceedings of the 2015 IEEE Global Communications Conference (GLOBECOM).

[14] Al-Talabani A., Nallanathan A., Nguyen H.X. Enhancing Secrecy Rate in Cognitive Radio via Game Theory. Proceedings of the 2015 IEEE Global Communications Conference (GLOBECOM).

[15] Talabani A., Deng Y., Nallanathan A., Nguyen H.X. (2016). Enhancing Secrecy Rate in Cognitive Radio Networks via Stackelberg Game. IEEE Trans. Commun..

[16] Talabani A., Deng Y., Nallanathan A., Nguyen H.X. (2016). Enhancing Secrecy Rate in Cognitive Radio Networks via Multilevel Stackelberg Game. IEEE Commun. Lett..

[17] Abdalzaher M.S., Seddik K., Muta O. (2017). Using Stackelberg game to enhance cognitive radio sensor networks security. IET Commun..

[18] Liu C., Xing S., Shen L. (2016). Dynamic hybrid-access control in multi-user and multi-femtocell networks via Stackelberg game competition. IET Commun..

[19] Sarma S., Kandhway K., Kuri J. (2016). Robust Energy Harvesting Based on a Stackelberg Game. IEEE Wirel. Commun. Lett..

[20] Zhang T., Chen W., Yang F. (2017). Balancing Delay and Energy Efficiency in Energy Harvesting Cognitive Radio Networks: A Stochastic Stackelberg Game Approach. IEEE Trans. Cognit. Commun. Netw..

[21] Xiong K., Fan P., Zhang C., Letaief K.B. (2015). Wireless Information and Energy Transfer for Two-Hop Non-Regenerative MIMO-OFDM Relay Networks. IEEE J. Sel. Areas Commun..

[22] Wang X., Liu J., Zhai C. (2017). Wireless power transfer based multi-pair two-way relaying with massive antennas. IEEE Trans. Wirel. Commun..

[23] Wang X., Zhai C. (2017). Simultaneous wireless information and power transfer for multi-user massive antenna-array systems. IEEE Trans. Commun..

[24] Khandaker M.R.A., Wong K.-K., Zhang Y., Zheng Z. (2017). Probabilistically Robust SWIPT for Secrecy MISOME Systems. IEEE Trans. Inf. Forensics Secur..

[25] Liu L., Zhang R., Chua K.-C. (2014). Secrecy Wireless Information and Power Transfer with MISO Beamforming. IEEE Trans. Signal Process..

[26] He B., Yang N., Yan S., Zhou X. (2016). Regularized Channel Inversion for Simultaneous Confidential Broadcasting and Power Transfer: A Large System Analysis. IEEE J. Sel. Top. Signal Process..

[27] Zhu Z., Chu Z., Wang N., Huang S., Wang Z., Lee I. (2017). Beamforming and Power Splitting Designs for AN-Aided Secure Multi-User MIMO SWIPT Systems. IEEE Trans. Inf. Forensics Secur..

[28] Zhou F., Li Z., Cheng J., Li Q., Si J. (2017). Robust AN-Aided Beamforming and Power Splitting Design for Secure MISO Cognitive Radio with SWIPT. IEEE Trans. Wirel. Commun..

[29] Osborne M.J., Rubenstein A. (1994). A Course in Game Theory.

[30] Haddad M., Hayely Y., Habachi O. (2015). Spectrum Coordination in Energy Efficient Cognitive Radio Networks. IEEE Trans. Veh. Technol..

[31] Rhee W., Cioffi J.M. Increase in capacity of multiuser OFDM system using dynamic subchannel allocation. Proceedings of the 2000 IEEE 51st Vehicular Technology Conference Proceedings (Cat. No. 00CH37026).

[32] Le T.A., Vien Q.T., Nguyen H.X., Kwan Ng D.W., Schober R. (2017). Robust Chance-Constrained Optimization for Power-Efficient and Secure SWIPT Systems. IEEE Trans. Green Commun. Netw..

[33] Zamanipour M. (2017). Probabilistic-based secrecy rate maximisation for MIMOME wiretap channels: Towards novel convexification procedures. Trans. Emerg. Telecommun. Technol..

[34] Ding H., Wan G., Zhou Y., Tang J., Zhou Z. (2017). Nonlinearity analysis based algorithm for indentifying machine settings in the tooth flank topography correction for hypoid gears. Mech. Mach. Theory.

[35] Bellavia S., Macconi M., Pieraccini S. (2012). Constrained dogleg methods for nonlinear systems with simple bounds. Comput. Optim. Appl..

[36] Grant M., Boyd S. (2009). CVX: Matlab Software for Disciplined Convex Programming.

[37] Fang B., Zhong W., Jin S., Qian Z., Shao W. (2016). Game-Theoretic Precoding for SWIPT in the DF-Based MIMO Relay Networks. IEEE Trans. Veh. Technol..

[38] Bannour A., Sacchi C., Sun Y. (2017). MIMO-OFDM Based Energy Harvesting Cooperative Communications Using Coalitional Game Algorithm. IEEE Trans. Veh. Technol..

[39] Adian M.G., Aghaeinia H. (2016). Low complexity resource allocation in MIMO-OFDM-based cooperative cognitive radio networks. Trans. Emerg. Telecommun. Technol..

[40] Boyd S., Vandenberghe L. (2004). Convex Optimization.

[41] Wang A., Cai Y., Yang W., Hou Z. A Stackelberg Security Game with Cooperative Jamming over a Multiuser OFDMA Network. Proceedings of the 2013 IEEE Wireless Communications and Networking Conference (WCNC).

[42] Hjørungnes A., Gesbert D. (2011). Complex-Valued Matrix Derivatives: With Applications in Signal Processing and Communications.

[43] Petersen K., Pedersen M. (2012). The Matrix Cookbook.

